# Zebularine protects against blood-brain-barrier (BBB) disruption through increasing the expression of zona occludens-1 (ZO-1) and vascular endothelial (VE)-cadherin

**DOI:** 10.1080/21655979.2021.2024323

**Published:** 2022-02-03

**Authors:** Xiangliang Zeng, Guohua He, Xirong Yang, Guoyao Xu, Yidan Tang, Hanwen Li, Bing Yu, Zhen Wang, Wei Xu, Kangping Song

**Affiliations:** a Department of Neurology, The First Affiliated Hospital of Hunan University of Medicine, Huaihua City, 418000, Hunan Province, China; b Department of Neurology, The affiliated Changsha Central Hospital, Hengyang Medical School, University of South China, Changsha 410004, Hunan Province, China

**Keywords:** Zebularine, stroke, blood-brain barrier, AMPKΑ, inflammation

## Abstract

Blood-brain-barrier (BBB) disruption is an important pathological characteristic of ischemic stroke (IS) and mainly results from dysfunction of brain vascular endothelial cells and tight junctions. Zebularine is a novel inhibitor of DNA methyltransferase (DNMT). Here, we assessed its effects on BBB disruption in IS. Firstly, we reported that Zebularine maintained BBB integrity in middle cerebral artery occlusion (MCAO) mice by increasing the expressions of zona occludens-1 (ZO-1) and vascular endothelial (VE)-cadherin. Importantly, we found that Zebularine reduced the production of pro-inflammatory cytokines, attenuated brain edema, and improved neurological deficits. In *in vitro* experiments, the bEnd.3 brain endothelial cells were exposed to oxygen and glucose deprivation/reoxygenation (OGD/R), and the protective effects of Zebularine were assessed. Our findings demonstrated that Zebularine prevented OGD/R-induced cytotoxicity by reducing the release of lactate dehydrogenase (LDH). Additionally, Zebularine protected bEnd.3 cells against OGD/R-induced hyper-permeability and reduction of trans-endothelial electrical resistance (TEER). Notably, we found that treatment with Zebularine activated the Adenosine 5ʹ-monophosphate (AMP)-activated protein kinase (AMPK) pathway by increasing the phosphorylation of adenosine monophosphate-activated protein kinase α (AMPKα). Blockage of AMPKα using its specific inhibitor compound C abolished the beneficial effects of Zebularine in mitigating endothelial hyper-permeability by reducing the expressions of ZO-1 and VE-cadherin. These findings suggest that the protective effects of Zebularine against OGD/R-induced endothelial hyper-permeability are mediated by the activation of AMPKα. In conclusion, our study sheds light on the potential application of Zebularine in the treatment of IS.

## Graphical abstract

**Figure:**
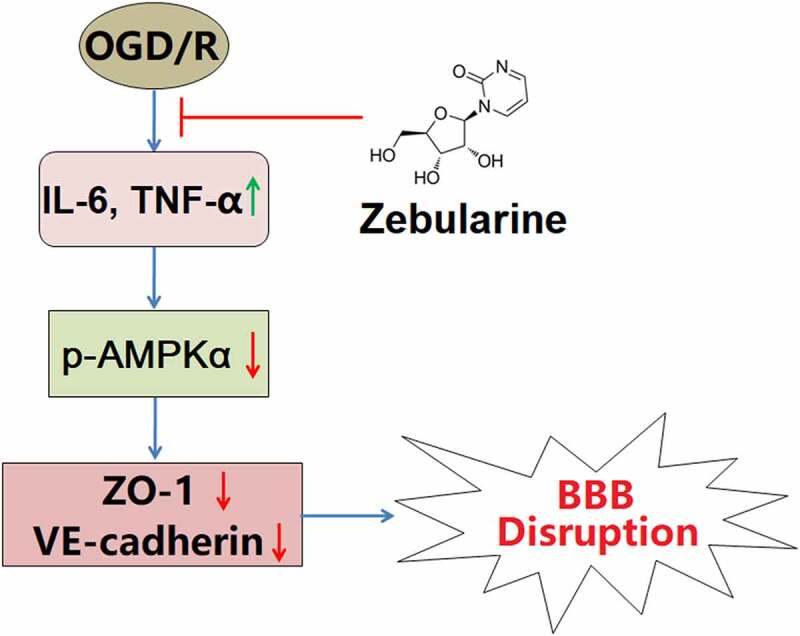

## Introduction

Ischemic stroke (IS) is a nervous system disease induced by endothelial dysfunction and autonomic nervous system dysfunction as a result of the narrowing or obstruction of the cerebral artery, and is characterized by emergent and focal neurological loss^1^. The clinical symptoms of IS include sudden fainting, side limb numbness, askew, and aphasia, which finally contribute to hemiplegia or even death [1]. The morbidity rate of IS significantly increases annually [2]. Thus, it is important to explore the underlying pathogenesis and effective therapies for the treatment of clinical IS. The BBB is a biochemical barrier located between the central nervous system (CNS) and circulatory system, it plays a critical role in maintaining CNS homeostasis and the normal function of neurons [3]. Damage to the BBB is reported to be an important pathological process involved in the pathogenesis of IS, which is the key inducer for the hemorrhagic transformation and adverse consequences of IS [4]. BBB dysfunction also results in the limited application of tissue plasminogen activator thrombolytic therapy for the treatment of IS [5]. The BBB mainly consists of tight junctions (TJs), endothelial cells, pericytes, astrocytes, neurons, and the basement membrane [6,7]. Endothelial cells are the first barrier of the BBB. When cerebral ischemia occurs, severe dysfunction of endothelial cells is induced by oxidative stress and inflammatory reactions, including changes to the cytoskeleton, degradation of TJs, and the dysfunction of translocators. Under the electronic microscope, the number of intracellular vesicles in endothelial cells increases greatly, and the process of transcytosis is enhanced 3 hours after cerebral ischemia sets in [8]. In addition, it is reported that autophagy in endothelial cells is also involved in the disruption of the BBB in IS. After oxygen and sugar deprivation, zona occludens-1 (ZO-1) is degraded, and the integrity of the BBB is disrupted by the activation of autophagy lysosomes in brain endothelial cells [9]. The degradation of TJ proteins is the main inducer of the increased BBB permeability in IS, which involves protein translocation, degradation, and down-regulation. It is reported that the expression levels of representative TJ proteins, such as ZO-1 and vascular endothelial (VE)-cadherin, are significantly declined in cerebral ischemia [10]. Therefore, it is vital to protect against endothelial dysfunction, and degradation of TJ proteins for the treatment of clinical IS.

**Figure 1.**
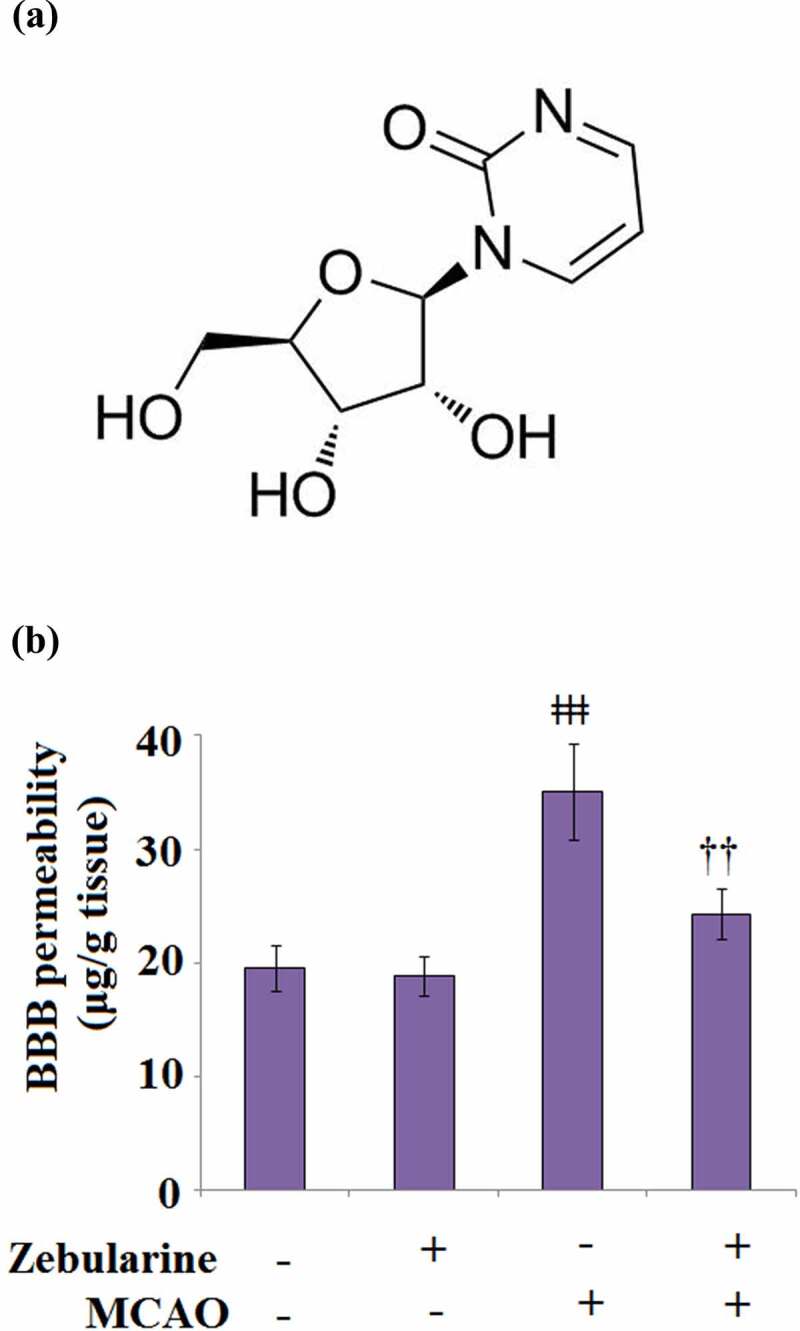
Zebularine maintained BBB integrity in MCAO mice. Mice were divided into 4 groups: the sham, sham+ Zebularine, MCAO, and MCAO+ Zebularine groups. (a). Molecular structure; (b). BBB permeability of Evan’s blue (ǂǂǂ, P < 0.001 vs. sham; ††, P < 0.01 vs. MCAO, N = 9).

Zebularine is a cytidine analogue that inhibits methylation, and its molecular structure (Figure 1a) is similar to that of 5-Azacytidin, which was first synthesized in 1961 as a bacteriostatic agent [11]. With the deepening of research, a significant inhibitory effect of Zebularine on cytidine deaminase has been widely reported [12]. Cheng reported that Zebularine exerted promising demethylation and anti-tumor effects with high selectivity to tumor tissues [13]. Recently, a significant neuroprotective effect of Zebularine has been widely reported [14]. However, the benefits of Zebularine in IS and BBB integrity remains unclear. Here, we investigated the pharmacological function of Zebularine in BBB disruption under the context of IS and explored the underlying mechanism.

## Material and methods

### Establishment of MCAO model in mice and grouping

Animal experiments were approved by the Ethics Committee of Hunan University of Medicine. C57BL/6 mice were obtained from the Laboratory Animal Center of Beijing University and were divided into 4 groups: the sham, sham+ Zebularine, MCAO, and MCAO+ Zebularine. The establishment of MCAO model in mice was conducted according to the procedure described previously [15]. Prior to MCAO modeling, animals in the sham and MCAO groups were administered with normal saline for 7 consecutive days. Mice in the sham+ Zebularine and the MCAO+ Zebularine groups were dosed with a daily administration of Zebularine at the dosage of 750 mg/kg for 7 consecutive days. In brief, after anesthetization on mice using tribromoethanol and atropine by intraperitoneal injection, mice were fixed in a supine position, and an incision was made in the middle of the neck, followed by stripping the right common carotid artery (CCA), external carotid artery (ECA), and internal carotid artery (ICA). The incision was sutured for the mice in the sham group. For mice in the MCAO groups, the blood flow was blocked using a caoutchouc, tied on the proximal part of the CCA and ECA, followed by inserting a fishing line into the ECA. Subsequently, the fishing line was further introduced to the initiation site of the middle cerebral artery until a slight resistance was felt, followed by being fixed using the surgical suture. Approximately 1 h later, the fishing line was taken out to recover the blood flow. Lastly, the incision was sutured for recovery.

### Evan’s blue for the determination of BBB permeability

After the modeling, mice were injected with 0.25 mL Evan’s blue solution dissolved in normal saline. After 24 hours, mice were anesthetized and myocardial perfused with normal saline, followed by isolating ischemic lateral forebrain and homogenate using 7.5% trichloroacetic acid. Subsequently, the homogenates were centrifugated at 12,000 × g and 4°C for 20 minutes, followed by collecting the supernatant for detection of absorbance at 620 nm. The levels of Evan’s blue were determined by drawing the standard curve using a series of concentrations of Evan’s blue [16].

### Enzyme-linked immunosorbent assay (ELISA)

The concentrations of tumor necrosis factor (TNF)-α and interleukin (IL)-6 in the serum were detected using ELISA assay. Briefly, supernatants were collected following centrifugation on the serum, followed by quantifying the concentrations of the proteins using the bicinchoninic acid assay. Subsequently, the samples were added to a flat-bottomed microtiter plate coated with primary antibodies, followed by incubation at 4°C overnight. Then, the samples were washed, and the HRP-linked secondary antibody was added for incubation for another 1 hour at room temperature, followed by adding 50 μL substrate. The optical density (OD) values at 450 nm were measured for the calculation of concentrations of TNF-α and IL-6 using the microplate spectrophotometer (Shenzhen Rongjin Technology Co. Ltd, Guangdong, China) after obtaining the standard curve with standard proteins.

### Real-time PCR analysis

Total RNA was extracted from the brain tissues from mice in each group using the Trizol solution, followed by determining the concentration of RNA and reverse transcription to obtain cDNAs. The specifically designed primers were used for the amplification of PCR using the cDNAs as the template. Lastly, the 2^−ΔΔCt^ method was used for determining the level of target genes after normalization to the glyceraldehyde-3-phosphate dehydrogenase (GAPDH) gene.

### Immunostaining of tight junction proteins

The brain tissues were extracted from mice in each group and were fixed using formalin, followed by being embedded with paraffin and sectioned. After being baked in a 65°C oven for 2 hours, the sections were mixed with xylene for 10 minutes, followed by being placed in 100%, 95%, 80% ethanol, and water for 5 minutes, successively. Sections were then placed in a citric acid buffer, and 3% fresh hydrogen peroxide was added, followed by incubation with 5% BSA. Subsequently, the sections were incubated with the primary antibody against ZO-1 (1:200, Abcam, Cambridge, UK) and VE-cadherin (1:200, Abcam, Cambridge, UK) overnight at 4°C, followed by 3 washes and incubation with the fluorescein 5-isothiocyanate (FITC)-labeled secondary antibody (1:100, Abcam, Cambridge, UK) at 37°C for 30 minutes. Lastly, images were taken using fluorescent microscopy (Olympus, Tokyo, Japan) [17].

### Measurement of brain water content

Briefly, after execution, the brain tissues were isolated from each animal, and the wet weights of the brain tissues were recorded. Subsequently, the brain tissues were dried in the oven to lose water, followed by recording the weight of the dry brain tissues. Lastly, the brain water content was calculated according to the following formula: brain water content (%) = (wet weight−dry weight)/wet weight×100%.

### Neurological score

Neurological deficits were scored 72 hours post-MCAO to estimate the degree of severity of the injury using the methods described previously [18]. A 6-point scale was used.

### Cells and OGD/R

The bEnd.3 brain endothelial cells were obtained from Fenghui Biological Co. Ltd (Changsha, China) and cultured in Dulbecco’s Modified Eagle Medium (DMEM) supplemented with 10% fetal bovine serum (FBS), streptomycin, and penicillin at 37°C and 5% CO_2_. For the induction of OGD/R *in vitro* model, bEnd.3 brain endothelial cells were cultured with deoxygenated medium under hypoxic conditions (1% O_2_, 5% CO_2_, 94% N_2_) in an air-tight incubator for 6 hours and subsequently exposed to reperfusion medium (21% O_2_, 5% CO_2_) at 37°C for 24 hours.

### Lactate dehydrogenase (LDH) release assay

After centrifugation at 300 × g for 10 **minutes**, the supernatant in the endothelial cells was collected, followed by adding the LDH substrate solution. After incubation at 37°C for 30 **minutes**, a microplate reader (Shenzhen Rongjin Technology Co. Ltd, Guangdong, China) was used for the detection of the level of released LDH at 440 nm.

### Fluorescein isothiocyanate (FITC)-dextran assay

The paracellular permeability of the bEnd.3 brain endothelial monolayers were measured by assessing the permeation of FITC-dextran across confluent cells grown on Trans-well filters. Cells were seeded at a density of 2 × 10^4^ cells/well in 200 μL medium onto 24-well Transwell chambers on Transwell inserts (6.5 mm diameter, 0.4 μm pore size; Corning, USA). The cell monolayer normally reaches confluency after 2 days. Cells were exposed to OGD/R with Zebularine (20 μM) for 24 hours. 500 μg/mL FITC-dextran (40 KDa) were then added to the apical compartment of the chamber. 2 hours later, 200 μL of the samples were collected from the lower chamber. The absorbance of the collected samples was recorded (Excitation: 485 nm; Emission: 528 nm) with a microplate reader (Molecular Devices, USA).

### Trans-endothelial electrical resistance (TEER)

2 × 10^4^ endothelial cells were seeded on the apical trans-well chamber (6.5 mm diameter, 0.4 μm pore size; Corning, USA) for 48 hours, followed by detecting the TJ function by TEER according to the method described previously [19] using the Millicell-ERS equipment. The values of TEER were calculated as Ωcm2 by multiplying the surface area of the trans-well insert.

### Western blot

Total proteins were extracted from endothelial cells using the radio-immunoprecipitation assay (RIPA) extraction reagents (ATTO, New York, USA), and the bicinchoninic acid assay was used to quantify the concentration of the total proteins, followed by loading 30 μg denatured protein into the sodium dodecyl sulfate-polyacrylamide gel electrophoresis (SDS-PAGE). Subsequently, the proteins were electro-transferred to PVDF membranes for 90 minutes, followed by being blocked using 5% BSA. After 3 washes, the PVDF membrane was incubated with the primary antibody against ZO-1 (1:1000, Santa Cruz, USA), VE-cadherin (1:1000, Santa Cruz, USA), p-AMPKα (1:1000, Santa Cruz, USA), AMPKα (1:1000, Santa Cruz, USA), and β-actin (1:1000, Santa Cruz, USA), followed by several washes and incubated with secondary antibody (1:2000, Santa Cruz, USA) for 2 hours at room temperature. Lastly, an enhanced chemiluminescent detection system (Beyotime Biotechnology, Shanghai, China) was used to visualize the immunoreactive proteins on the membrane.

## Statistical analysis

All data in the present study were reported as mean ± standard deviation (S.D.) and ANOVA test was used for the comparison among groups. All data were analyzed using tGraphPad Prism 8, and p < 0.05 was regarded as a significant difference.

## Results

To investigate the potential benefits of Zebularine against BBB disruption, we established both the MCAO mice model and the brain endothelial cells model. In the MCAO model, we examined the effects of Zebularine on BBB integrity, expressions of ZO-1, VE-cadherin, and pro-inflammatory cytokines, as well as brain edema and neurological deficits. In endothelial cell models, we tested for the effects of Zebularine on LDH release, expressions of ZO-1 and VE-cadherin, alteration of endothelial permeability and TEER. Mechanistically, we further assessed the involvement of AMPKα in mediating the protective effects of Zebularine.

### Zebularine maintained BBB integrity in MCAO mice

To determine the promising protective effect of Zebularine against BBB disruption in MCAO mice, mice were administered with Zebularine for 7 consecutive days. As shown in Figure 1b, no significant difference was observed between the sham and sham + Zebularine groups. Compared to the sham group, the BBB permeability was significantly elevated from 19.6 μg/g tissue to 35.1 μg/g tissue in the brain tissues of MCAO mice, then dramatically declined to 24.3 μg/g tissue in the MCAO mice treated with Zebularine, indicating an inhibitory effect of Zebularine on the permeability of the disrupted BBB.

### Zebularine restored ZO-1 and VE-cadherin expression in the brains of MCAO mice

**Figure 2.**
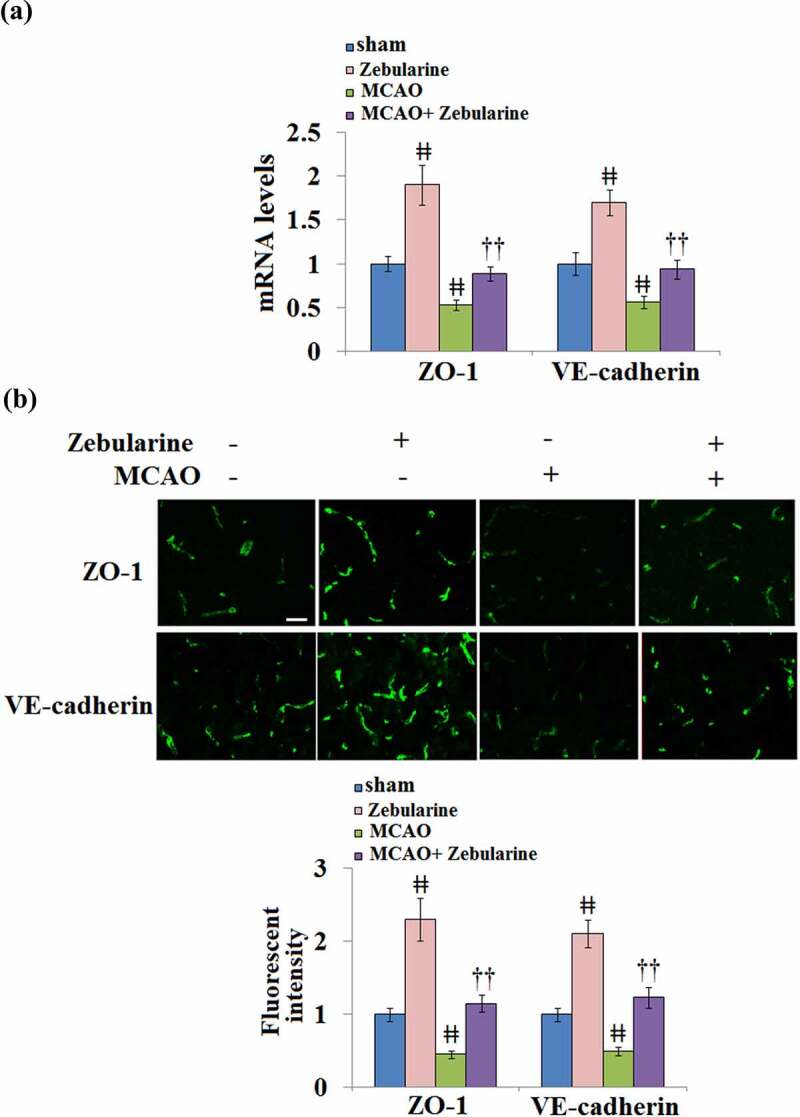
Zebularine restored ZO-1 and VE-cadherin expression in the brains of MCAO mice. Mice were divided into 4 groups: the sham, sham+ Zebularine, MCAO, and MCAO+ Zebularine groups. (a). ZO-1 and VE-cadherin mRNA expression in brain cortex; (b). Immunostaining of ZO-1 and VE-cadherin in brain cortex, Scale bar, 100 μm (ǂǂ, P < 0.01 vs. sham; ††, P < 0.01 vs. MCAO, N = 9).

The expression levels of TJ proteins in the brain tissues were further investigated. As shown in Figure 2a and B, we found that the levels of ZO-1 and VE-cadherin were significantly elevated in the sham + Zebularine group and suppressed in the MCAO group. Compared to MCAO mice, ZO-1 and VE-cadherin were significantly upregulated in the MCAO mice treated with Zebularine, displaying a promising regulatory effect of Zebularine on the TJ function.

### Zebularine suppressed the secretion of pro-inflammatory cytokines in MCAO mice

**Figure 3.**
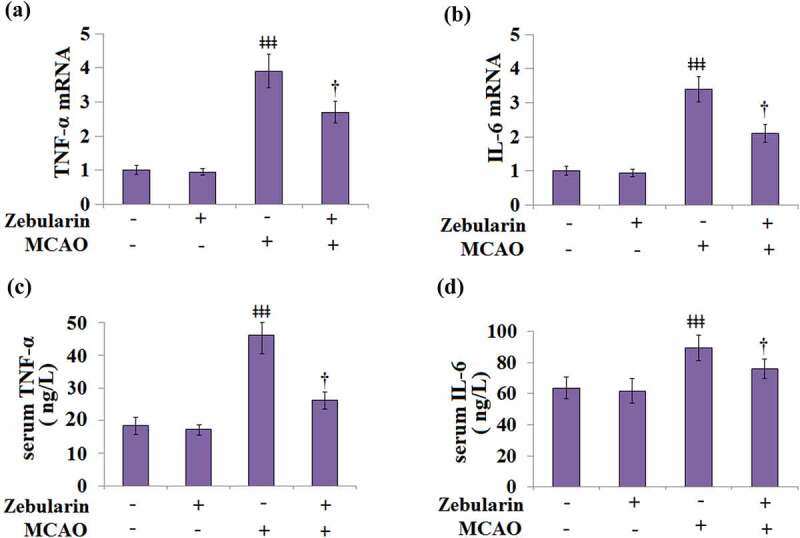
Zebularine suppressed secretion of pro-inflammatory cytokines in MCAO mice. Mice were divided into 4 groups: the sham, sham+ Zebularine, MCAO, and MCAO+ Zebularine groups. (a) TNF-α mRNA expression in brain cortex; (b). IL-6 mRNA expression in brain cortex; (c). Levels of serum TNF-α; (d). Levels of serum IL-6 (ǂǂǂ, P < 0.001 vs. sham; †, P < 0.05 vs. MCAO, N = 9).

Firstly, we measured the mRNA levels of the pro-inflammatory cytokines TNF-α and IL-6 in the brain cortices of the experimental mice. Results in Figure 3a and b indicate that the mRNA levels of TNF-α and IL-6 were significantly increased in MCAO mice, then inhibited by Zebularine dose-dependently. ELISA was used to measure the levels of inflammatory factors in the serum. As shown in Figure 3c, the concentrations of TNF-α in the sham and sham + Zebularine groups were 18.5 and 17.3 ng/L, respectively, then significantly increased to 46.2 ng/L in the sera of MCAO mice. After treatment with Zebularine, the production of TNF-α in the MCAO mice was significantly declined to 26.3 ng/L. In addition, the secretion of IL-6 (Figure 3d) in the sera of animals from the sham and sham + Zebularine groups was 63.7 and 61.6 ng/mL, respectively, which was dramatically elevated to 89.5 ng/mL in the MCAO group. Following treatment with Zebularine, the concentration of IL-6 was reversed to 75.9 ng/L in the MCAO mice. These data indicate that the severe inflammation in MCAO mice was significantly alleviated by Zebularine.

### Zebularine ameliorated brain edema and improved neurological deficits in MCAO mice

**Figure 4.**
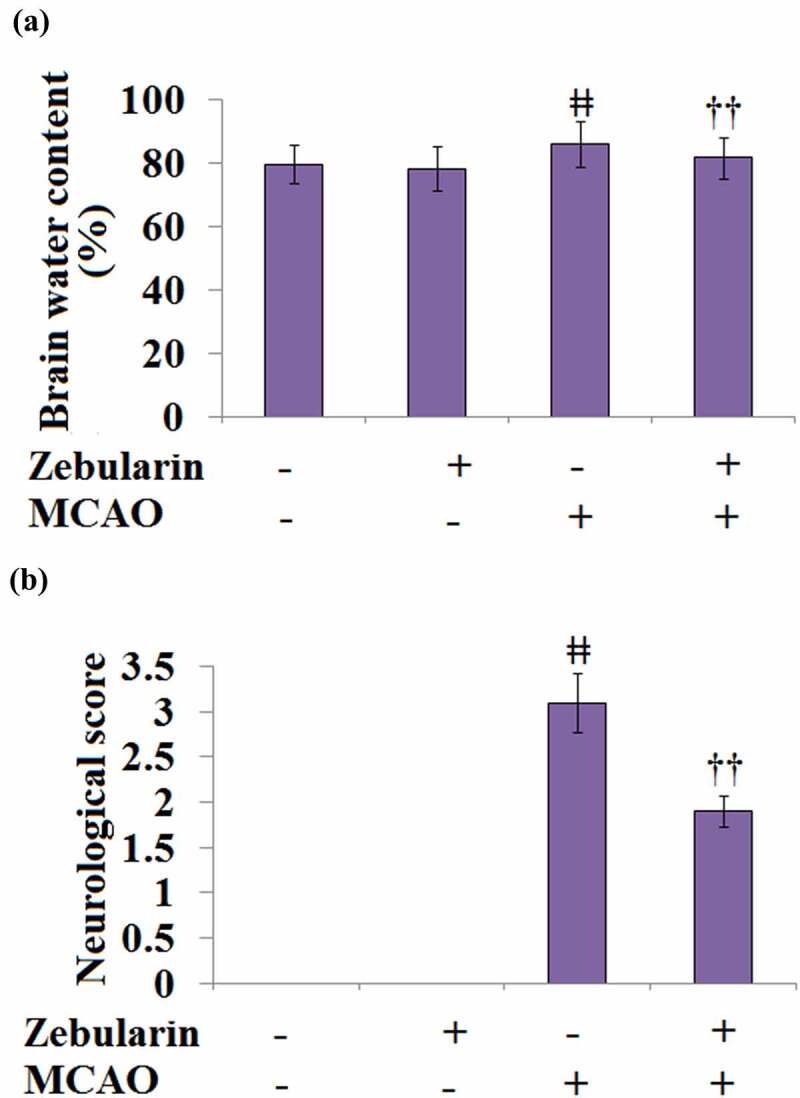
Zebularine ameliorated brain edema and improved neurological deficits in MCAO mice. Mice were divided into 4 groups: the sham, sham+ Zebularine, MCAO, and MCAO+ Zebularine groups. (a). Brain water content; (b). Neurological score (ǂǂ, P < 0.01 vs. sham; ††, P < 0.01 vs. MCAO, N = 9).

As shown in Figure 4a, the brain water content in the sham and the sham + Zebularine groups was 79.7% and 78.3%, respectively, then significantly promoted to 85.9% in the MCAO mice. The brain water content was then significantly decreased to 81.7% by the treatment with Zebularine.

Neurological deficits were scored at 72 hours post-MCAO to estimate the degree of severity of the injury. The results of the neurological score are shown in Figure 4b. All the neurological scores in the sham and the sham + Zebularine groups were recorded as 0 but significantly promoted to 3.1 in the MCAO mice. After the treatment with Zebularine, the neurological score was dramatically decreased to 1.9. These data reveal the potential neuroprotective effects of Zebularine.

### Zebularine prevented OGD/R-stimulated release of LDH in endothelial cells

**Figure 5.**
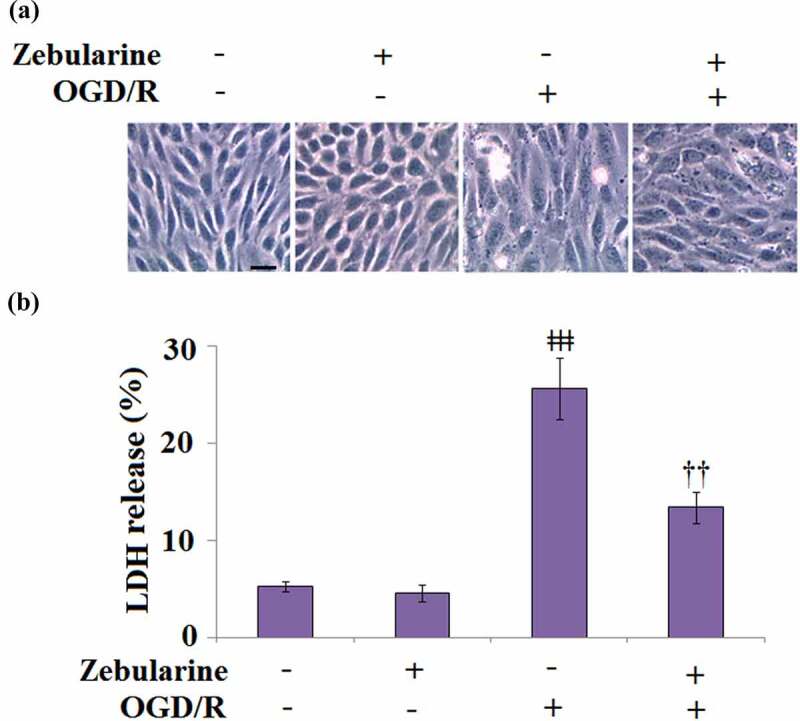
Zebularine prevented OGD/R-induced release of LDH in Bend.3 brain endothelial cells. Cells were exposed to OGD/R with Zebularine (20 μM). (a). Morphology of Bend.3 brain endothelial cells; Scale bar, 50 μm; (b). Release of LDH was assayed (ǂǂǂ, P < 0.001 vs. sham; ††, P < 0.01 vs. OGD/R, N = 5).

To explore the potential mechanism underlying the neuroprotective effects of Zebularine, bEnd.3 cells were exposed to OGD/R in the presence of 20 μM Zebularine. The morphology of cells is shown in Figure 5a. Compared to the control, no significant difference was observed on LDH release (Figure 5b) in the Zebularine group. However, the LDH release was significantly promoted from 5.3% to 25.6% in the endothelial cells under OGD/R conditions but dramatically reversed to 13.4% by the introduction of Zebularine. These data indicate that Zebularine protected endothelial cells from OGD/R- induced dysfunction.

### Zebularine mitigated alteration of endothelial permeability and TEER in OGD/R-challenged bEnd.3 brain endothelial cells

**Figure 6.**
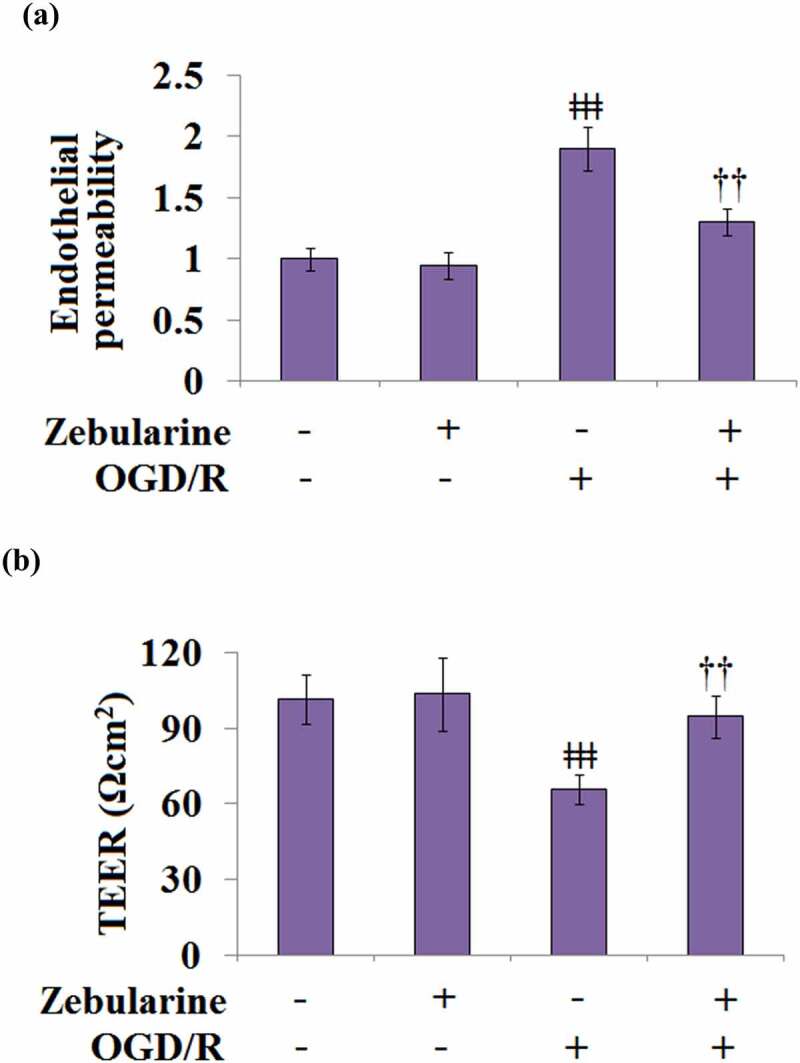
Zebularine ameliorated alteration of endothelial permeability and TEER in OGD/R- challenged Bend.3 brain endothelial cells. (a). Endothelial permeability; (b). The trans-endothelial electrical resistance (TEER) (ǂǂǂ, P < 0.001 vs. sham; ††, P < 0.01 vs. OGD/R, N = 5).

We further investigated the effects of Zebularine on the damaged endothelial monolayer established by endothelial cells. No significant difference was observed in the endothelial permeability between the control and Zebularine groups, whereas it was significantly increased in the OGD/R group (Figure 6a). After the treatment with Zebularine, the OGD/R- induced endothelial permeability was dramatically suppressed. In addition, TEER in the control and Zebularine groups was 101.5 and 103.6 Ωcm^2^, respectively, then significantly declined to 65.9 Ωcm^2^ in the OGD/R group. Following Zebularine treatment, TEER was greatly reversed to 94.6 Ωcm^2^ (Figure 6b). These results indicate that the elevated endothelial permeability caused by OGD/R was obviously ameliorated by Zebularine.

### Zebularine restored the expressions of ZO-1 and VE-cadherin in OGD/R- challenged bEnd.3 brain endothelial cells

**Figure 7.**
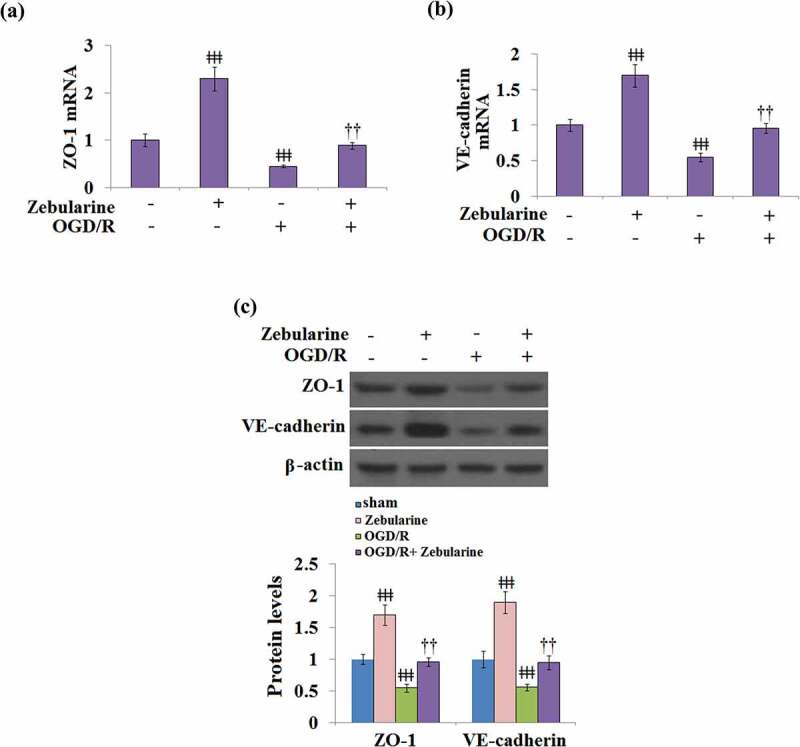
Zebularine restored the expression of ZO-1 and VE-cadherin in OGD/R- challenged Bend.3 brain endothelial cells. (a). mRNA of ZO-1 (; (b). mRNA of VE-cadherin; (c). Protein of ZO-1 and VE-cadherin (ǂǂǂ, P < 0.001 vs. sham; ††, P < 0.01 vs. OGD/R, N = 5).

We further explored the impact of Zebularine on TJ proteins in endothelial cells. As shown in Figure 7a-C, compared to the control, the expressions of ZO-1 and VE-cadherin were significantly elevated in the Zebularine group and suppressed in the OGD/R group, then later greatly reversed by the treatment with Zebularine in the bEnd.3 cells under OGD/R conditions.

### The effects of Zebularine on the expressions of ZO-1 and VE-cadherin and endothelial permeability are mediated by AMPKα

**Figure 8.**
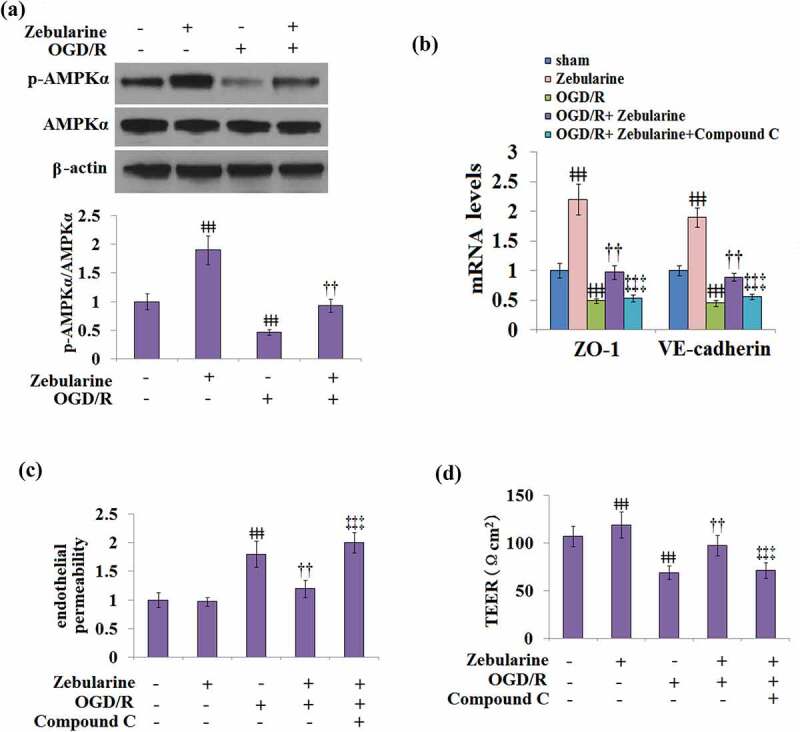
The effects of Zebularine in the expression of ZO-1 and VE-cadherin and endothelial permeability are mediated by AMPKα. (a). Cells were exposed to OGD/R with Zebularine. P-AMPKα and total AMPKα were measured; (b-d). Cells were exposed to OGD/R with Zebularine in the presence of Compound C. mRNA of ZO-1 and VE-cadherin, endothelial permeability, and TEER were measured (ǂǂǂ, P < 0.001 vs. sham; ††, P < 0.01 vs. OGD/R; ‡‡‡, OGD/R+ Compound C group, N = 5).

As shown in Figure 8a-B, the expression of AMPKα remained unchanged in all groups. Compared to the control, the expression of p-AMPKα/AMPKα was pronouncedly elevated in the Zebularine group and declined in the OGD/R group, which was later mitigated by the introduction of Zebularine in cells under the OGD/R state. To further verify the involvement of the AMPKα pathway in the mechanism of Zebularine, cells were exposed to OGD/R with Zebularine in the presence of compound C, an AMPKα pathway inhibitor. As shown in Figure 8c, compared to the OGD/R group, ZO-1 and VE-cadherin were significantly upregulated by the introduction of Zebularine, which was dramatically reversed by the co-treatment with compound C. In addition, compared to the OGD/R group, the endothelial permeability was greatly decreased by the incubation with Zebularine, then significantly elevated by the co-administration of compound C. In endothelial cells stimulated with OGD/R, TEER was dramatically promoted from 69.2 Ωcm^2^ to 97.5 Ωcm^2^ by the introduction of Zebularine, which was reversed to 71.2 Ωcm^2^ by the co-treatment with compound C (Figure 8d). These data indicate that Zebularine might protect endothelial permeability and TJ function by activating the AMPKα pathway.

## Discussion

Under a normal physiological state, a majority of molecules are not allowed past the BBB by the adjacent endothelial cells and TJs. TJ proteins that play important roles in maintaining the integrity of the BBB include Claudins, which connect to the cytoskeleton through the intracellular zonula occludens family (ZO) [20]. Under the pathological state of IS, significant local energy impairment and excessive production of oxidative stress products are induced by glucose and oxygen deprivation, and the subsequent reperfusion injury [21], all contributing to the disruption of BBB components and finally, the destruction of its integrity. The dysfunction of endothelial cells is one of the main inducers for the disruption of the BBB during IS. The shortage in ATP supply and excessive production of reactive oxygen species (ROS) are induced under the IS state, contributing to the contraction of actin protein, the increase of cytoskeletal tension, and impairment of cellular morphology in the endothelial cells. As a consequence, the TJ structures among brain vascular endothelial cells are damaged, and the permeability of the BBB increased [22]. Here, we established the *in vivo* MCAO mice models, verified by the increased BBB permeability and deteriorated neurological deficits, accompanied by the excessive production of inflammatory factors and downregulation of TJ proteins. These observations were consistent with previous studies [23,24]. After 7-days treatment with Zebularine, the BBB permeability, peripheral inflammation, neurological deficits, and TJs function were obviously mitigated, indicating a promising protective property of Zebularine on brain injury during IS. In addition, the establishment of the *in vitro* OGD/R models in mice brain endothelial cells was verified by the increased LDH release and endothelial permeability. After the incubation with Zebularine, the endothelial permeability, cell viability, and secretion of TJ proteins were dramatically mitigated, indicating a promising beneficial effect of Zebularine on endothelial dysfunction. Interestingly, in both the animal and the cellular experiments, no significant impact of Zebularine was observed on the permeability under normal physiological states (animals in the sham group and cells untreated with OGD/R), indicating that the pharmacological safety of Zebularine is relatively high due to specific effects under pathological states, which requires further verification in our future work.

AMPK (AMP-activated protein kinase) is a nutrient-sensitive kinase that is composed of an α catalytic subunit and non-catalytic subunits (β and γ). AMPK is widely expressed in eukaryotes and is an important factor involved in angiogenesis post-ischemia. The expression level of VEGF is elevated by the activation of the AMPK pathway [25]. In endothelial cells, ROS-related endoplasmic reticulum stress and inflammatory reactions are inhibited by the activation of AMPK [26]. It has been recently reported in a cerebral ischemic model, the expression levels of TNFα, caspase-3 and Bax/Bcl-2 were down-regulated by AMPK, contributing to the alleviation of apoptosis in neurons [27]. In the rat cerebral ischemic model, inflammation and oxidative stress were significantly ameliorated by the activation of AMPK, which decreased the cerebral infarct volume, alleviated the apoptosis of neurons, and ameliorated the symptoms of neurological deficits [28]. Here, we report that the expression of p-AMPK-α in endothelial cells under OGD/R conditions was dramatically elevated by Zebularine, indicating that Zebularine activated the AMPK-α pathway in endothelial cells. For further verification, we found that the inhibitor of AMPK-α abolished the protective effect of Zebularine on endothelial permeability and TJ function, revealing that Zebularine might exert its protective property in IS by activating the AMPK signaling pathway. In our future work, more functional assays will be introduced to highlight the protective effects of Zebularine on injured endothelial cells, such as detecting the biomarkers of oxidative stress. In addition, the direct target of Zebularine for the regulation of the AMPK signaling pathway will be explored to better understand the underlying mechanism of Zebularine.

## Conclusion

To sum up, our data reveal that Zebularine protected against BBB disruption through increasing the expressions of ZO-1 and VE-cadherin. We also provide evidence that the protective effects of Zebularine were mediated by activating the AMPK signaling pathway. Our study sheds a light on the potential application of Zebularine in the prevention and treatment of IS.

## Data Availability

Experimental data are available on reasonable request to the corresponding author.
